# Inferring personal economic status from social network location

**DOI:** 10.1038/ncomms15227

**Published:** 2017-05-16

**Authors:** Shaojun Luo, Flaviano Morone, Carlos Sarraute, Matías Travizano, Hernán A. Makse

**Affiliations:** 1 Levich Institute and Department of Physics, City College of New York, New York, New York 10031, USA; 2 Grandata Labs, 550 15th Street, San Francisco, California 94103, USA

## Abstract

It is commonly believed that patterns of social ties affect individuals' economic status. Here we translate this concept into an operational definition at the network level, which allows us to infer the economic well-being of individuals through a measure of their location and influence in the social network. We analyse two large-scale sources: telecommunications and financial data of a whole country's population. Our results show that an individual's location, measured as the optimal collective influence to the structural integrity of the social network, is highly correlated with personal economic status. The observed social network patterns of influence mimic the patterns of economic inequality. For pragmatic use and validation, we carry out a marketing campaign that shows a threefold increase in response rate by targeting individuals identified by our social network metrics as compared to random targeting. Our strategy can also be useful in maximizing the effects of large-scale economic stimulus policies.

## Abstract (web summary)

It is believed that patterns of social ties are related to individuals' financial status. Here the authors substantiate this concept by quantitatively demonstrating that a measure of an individual's location and influence within their social network can be used to infer their economic wellness.

The long-standing problem of how the network of social contacts^1^,^2^,^3^ influences the economic status of individuals has drawn large attention due to its importance in a diversity of socioeconomic issues ranging from policy to marketing^4^,^5^,^6^,^7^. Theoretical analyses have pointed to the importance of the social network in economic life^5^ as a medium to diffuse ideas^8^,^9^ through the effects of ‘structural holes'^10^ and ‘weak ties' in the network^4^. Likewise, research has recognized the positive economic effect of expanding an individual's contacts outside its own tightly connected social group^1^,^11^,^12^,^13^. While previous work has established the importance of social network influence to economic status, the problem of how to quantify such correspondence via social network centralities or metrics^3^,^14^ remains open.

Studies employing mobile phone communication data and other social indicators have found a variety of network effects on socioeconomic indicators such as job opportunities^15^,^16^, social mobility^17^,^18^,^19^, economic development^6^,^20^,^21^,^22^ and consumer behaviour^23^,^24^. Recent work also provides evidence of such effects on an individual's wealth, and highlights the need for better indicators^25^. Recently, a numerical study has tested the effect of network diversity on economic development^6^. This study analysed economic development defined at the community level. However, the question of how social network metrics may be used to infer financial status at the individual level—necessary, for instance, for micro-target marketing or social intervention campaigns—still remains unanswered. The difficulty arises, in part, due to the lack of empirical data combining an individual's financial information with the pattern of their social ties at the large-scale network level of the whole society.

In this work, we address this problem directly by combining two massively large data sets: a social network of the whole population of a Latin American country and financial banking data at the individual level. We discover that the optimality of an individual's location in the network, which is measured by the collective influence (CI) metric^26^, is highly correlated with the individual's economic status at the population level: the larger the CI, the higher the socioeconomic level. The goodness of fit of this correlation can be as high as *R*^2^=0.99 when age is also included. These results indicate that the location's optimality in the social network measured by the CI metric can accurately predict socioeconomic indicators at the personal level.

The top 1% of the economic stratum has precise network patterns of ties formation showing relatively low local connectivity surrounded by a hierarchy of hubs strategically located in spheres of influence of increasing size in the network. Such a pattern is not observed in the rest of the population, in particular, in the bottom 10% characterized by low values of CI. Thus, the influence measured from social network patterns mimics the inequality observed in economic status^27^.

We also find a high correlation between the link diversity of individuals and their financial status (*R*^2^=0.96), employing the analysis based on network location and age. Analysis of the covariance suggests that the effect of network influence is significant and independent from other factors. We validate these results by carrying out a targeted marketing campaign in which we compare the response rate for different groups of people with different network locations. By targeting the group with the top CI values, the response rate can reach as high as 1%; approximately three times the response rate found by random targeting and five times the response rate of the low CI people.

Thus, individuals with high socioeconomic status (top 1%) develop a very characteristic pattern of social ties as compared to the bottom 10%. While this result may be expected, it is remarkable that the difference in pattern of social interactions between the rich and the poor can be precisely captured by a network metric measuring their CI in the social network^26^. The top socioeconomic layer of society also represents the minimal set of people that provides integrity to the whole social network through their large CI. The fact that individuals of higher economic status are located in regions of large CI in the network elevates previous anecdotal evidence to a principle of network organization through the optimization of influence of affluent people affecting the structural integrity of the social network. At the same time, it suggests the emergence of the phenomenon of CI in society as the result of the optimization of socioeconomic interactions.

## Results

### Network construction

The social network is constructed from mobile (calls and SMS metadata) and residential communications data collected for a period of 122 days (Supplementary Note 1, aggregated data at kcorelab.com). The database contains 1.10 × 10^8^ phone users. After filtering the non-human active nodes by a machine-learned model trained on human natural communication behaviour (Supplementary Note 2, with Supplementary Figs 1–4), we construct a final network of 1.07 × 10^8^ nodes in a giant connected component made of 2.46 × 10^8^ links. The ties, or links, in the network correspond to phone call communications, since we expect that communication patterns are indicative of an individual's location in the social network^28^,^29^,^30^. The financial cost of using phone services makes it possible that there is a systematic bias in how much wealthy individuals use the phone services relative to people that have less money to spend on phone calls. Although the effect might be limited (Supplementary Note 1), we cannot rule out this possibility with the present data.

Financial status is obtained from the combined credit limit on credit cards assigned by banking institutions to each client. The credit limit is based on composite factors of income and credit history and therefore reflects the financial status of the individual (see discussion in Supplementary Note 1). The credit limit is pulled from an encrypted bank database and identified by the encrypted clients' phone numbers registered in the bank. Thus, we are able to precisely cross-correlate the financial information of an individual with their social location in the phone call network at the country level. There are 5.02 × 10^5^ bank clients who have been identified in the mobile network whose credit limit ranges from USD $50 to $3.5 × 10^5^ (converted from the country of study). Thus, the data sets are precisely connected providing an unprecedented opportunity to test the correlation between network location and financial status.

Despite the large scale of our data source, we note that working on a single specific country as in the present study is not enough to grant generality to our results. To test the general validity of the present results, access to other countries' whole-population-level communication and banking data sets would be needed. As more data sets become available, the generality of our results can be tested across different economic and social systems.

**Figure 1:**
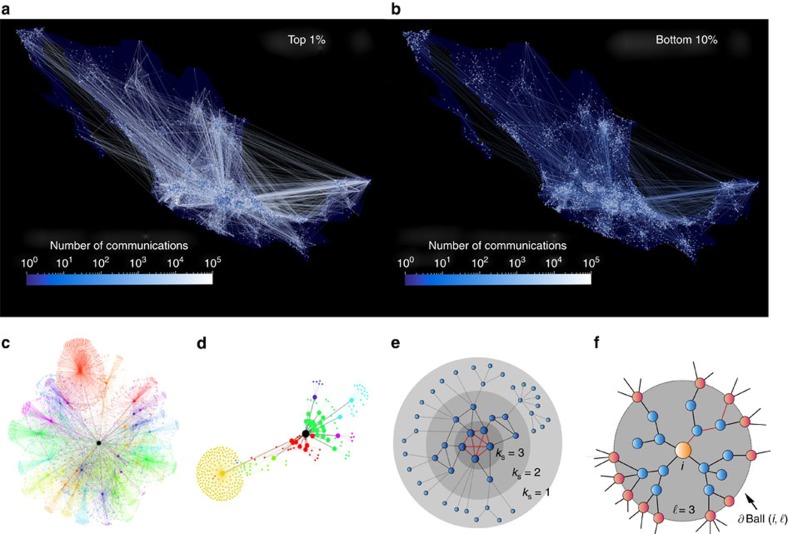
Patterns of network influence mimic patterns of income inequality. Visualization of communication activity of the population in **a** the top 1% (with credit limit larger than USD $25,000, converted, in the country of study) and (**b**) bottom 10% (with credit limit smaller than USD $600, converted) of total credit limit classes. Links are between bank clients who have registered their zip code. Resolution of both plots is 1,700 × 1,000. The number of bank clients inside each community is reflected by the size of the node. Average credit limit is denoted by a node's grayscale. The colour and thickness of the edges reflects the number of communication events between different communities. (**c**) Examples of the ego-network (extended to two layers) for an individual in the top 1% wealthy class and (**d**) an individual in the bottom 10% class. The networks show two distinct patterns of social ties according to high and low economic status: the former is characterized by large CI, the latter by low CI. (**e**) Schematic representation of a network under k-shell decomposition^33^. (**f**) Example of the calculation of CI. The CI Ball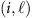 of radius 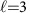 around node *i* is the set of nodes contained inside the sphere and *∂*Ball is the set of nodes on the boundary (brown). CI is the degree-minus-one of the central node times the sum of the degree-minus-one of the nodes at the boundary of the sphere of influence.

Figure 1a,b shows the communication patterns geolocalized across the country of individuals in the top 1% and bottom 10% of credit limits, respectively. The inequality in the patterns of communication between the top economic class and the lowest is striking and mimics the economic inequality at the country level^27^. It is visually apparent that the top 1% (accounting for 45.2% of the total credit in the country) displays a completely different pattern of communication than the bottom 10%; the former is characterized by more active and diverse links, especially connecting remote locations and communicating with other equally affluent people. Further results using entropy analysis also suggest that the network structure may be significantly different between the people in the top and bottom quantile rankings of credit limit (Supplementary Note 3; Supplementary Table 1). Particular examples of the extended ego-networks for two individuals (with same number of ties) ranking in the top 1% and bottom 10% provide a zoomed-in picture of such differences (Fig. 1c,d, respectively). The wealthiest 1-percenters have higher diversity in mobile contacts and are centrally located, surrounded by other highly connected people (network hubs). On the other hand, the poorest individuals have low contact diversity and are weakly connected to fewer hubs. The crux of the matter is to find a reliable social network metric to quantify this visual difference in the patterns of network structure between the rich and the poor, as we show next.

### Network influence and financial status

Many metrics or centralities have been considered to characterize the influence or importance of nodes in a network^3^,^14^,^31^. Here we consider only those centralities that can be scaled up to the large network size considered here (Fig. 1e,f; Supplementary Note 4): (a) degree centrality *k*_*i*_ (number of ties of individual *i*) is one of the simplest^3^, (b) PageRank, of Google fame^32^, is an eigenvector centrality that includes the importance of not only the degree, but also the nearest neighbors, (c) the k-shell index *k*_s_ of a node (Fig. 1e), that is, the location of the shell obtained by iteratively pruning all nodes with degree *k*<*k*_s_ (ref. 33), and (d) the CI of a node with degree *k*_*i*_ (Fig. 1f) in a sphere of influence of size 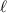 defined by the frontier of the influence ball 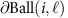, and predicted to be 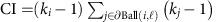 by optimal percolation theory^26^. As opposed to the other heuristic centralities, CI is derived from the theory of maximization of influence in the network^34^. The top CI nodes are thus identified as top influencers or superspreaders of information, and they are so by positioning themselves at strategic locations at the centre of spheres surrounded by hubs hierarchically placed at distances 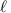 (Fig. 1d). These collective influencers also constitute an optimal set that provides integrity to the social fabric: they are the smallest number of people that, upon leaving the network (a process mathematically known as optimal percolation^26^), would disintegrate the network into small disconnected pieces.

**Figure 2:**
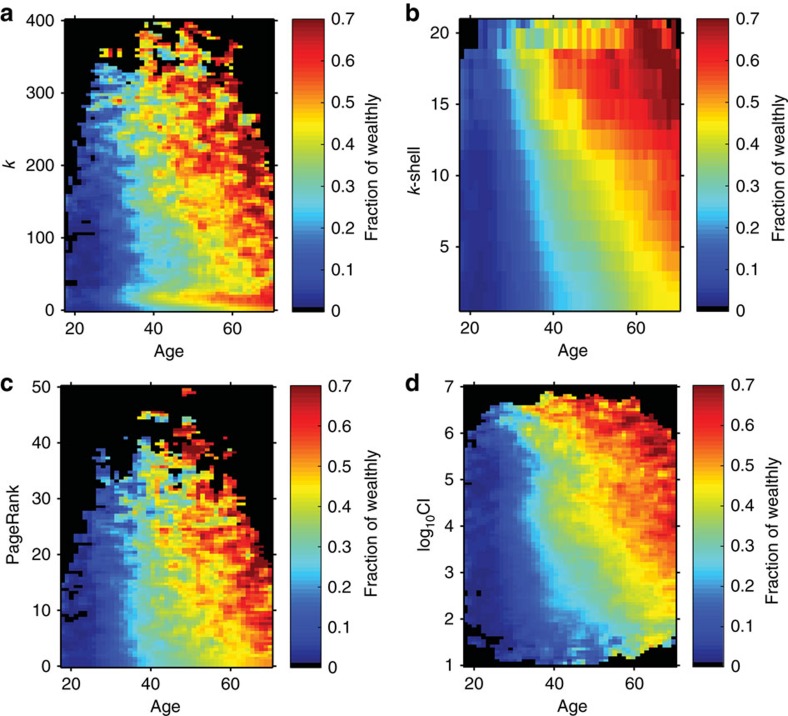
Fraction of wealthy individuals versus age and network metrics. Correlation between the fraction of wealthy individuals versus age and (**a**) degree *k* (*R*^2^=0.92), (**b**) k-shell (*R*^2^=0.96), (**c**) PageRank (*R*^2^=0.96) and (**d**) log_10_CI (*R*^2^=0.93). Only those groups with population >20 are shown in the plot. The four metrics correlate well with financial status when considered with age. Further correlations are studied in Supplementary Note 6, indicating that CI could be considered as the most convenient metric out of the four due to its high resolution.

By definition, all of the metrics have similarities (for example, they are proportional to *k*, and PageRank and CI are based on the largest eigenvalues of the adjacency and non-backtracking matrices, respectively^26^), and indeed, we find that their values in the phone communications network are correlated (Supplementary Table 2). More interestingly, Fig. 2 provides evidence of correlation of the four network metrics with financial status (ranked credit limit) when we control for age, indicating that the network location correlates with financial status. In this figure, we plot the fraction of wealthy individuals (defined as top 4th quantile, equivalent to a credit limit greater than USD $4,000; see Supplementary Note 5 for details about validation methods and ref. 30) in a sampling grid for a given value of age and social metric as indicated.

While all of the social metrics show correlations with financial status when considered with age (Fig. 2), the question remains of which metric is the most efficient predictor. Strong correlations with economic wellness are observed for the feature pairs (age, k-shell; *R*^2^=0.96, Fig. 2b) and (age, CI; *R*^2^=0.93, Fig. 2d). Supplementary Note 6 (Supplementary Figs 7–9) provides further comparison when considering the metrics alone, indicating that k-shell and CI better capture the correlation with credit limit. Between these two metrics, CI guarantees a requirement for both strong correlation and sufficient resolution. K-shell cannot capture further details due to its limitation of values (k-shell ranges from 1 to 23, dividing the whole population into this small number of shells with a typical shell containing tens of millions of people), while CI spans over seven orders of magnitude; see Supplementary Fig. 5. This high resolution implies that CI is a more accurate social signature for the financial status of the individuals. According to its definition (Fig. 1d), a top CI node is a moderate-to-strong hub surrounded by other hubs hierarchically placed at distance 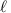. However, we emphasize that CI is just a useful strategy for the reasons shown above, and by no means the only or best strategy to correlate the wealth of individuals and their network influence.

While the theory behind CI is a global maximization of influence, CI represents the local approximation to this global optimization. Thus, CI represents a balance between a global optimization and its local approximation, taking into account the first 2 or 3 layers of neighbours via the parameter 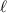, which represents the size of the sphere of influence used to define the importance of a node, Fig. 1d. By changing 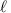, we discover that CI with 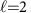 is sufficient to capture the correlation between network influence and wealth (Supplementary Fig. 10).

**Figure 3:**
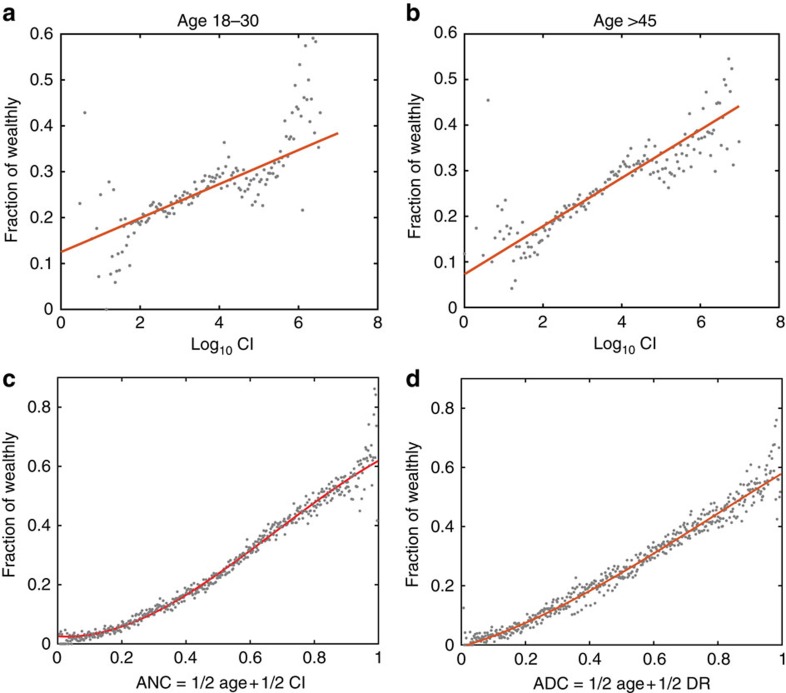
Fraction of wealthy individuals over different age and composite ranking groups. Correlation between the fraction of wealthy individuals as given by the top 25% credit limit and CI in different age groups of (**a**) 18–30 and (**b**) >45. Correlations between top economic status and large CI as determined by CI values in different ages are significant in all age groups, while the slope of the linear regression is larger in the older group (0.053 compared to 0.037). (**c**) Age-network composite ranking ANC=1/2 Age+1/2 CI, and (**d**) age-diversity composite ranking ADC=1/2 Age+1/2 DR. By combining the network metrics with age into a composite index, the chance to identify people of high financial status reaches ∼70% for high values of the composite. Both *R*^2^'s show a high level of correlation (*R*^2^=0.99 and 0.96 for ANC and ADC, respectively), making both composites good predictors of wealth in practical applications.

To track the effect of CI independently of age, we investigate the effects of CI inside two specific age groups in Fig. 3a,b. In both age groups, high CI is always accompanied by a higher population of wealthy people. A relatively smaller slope in age group <30 suggests that the CI network effect is more sensitive for older people with more mature and stable economic levels, than for younger people (Supplementary Fig. 6). When we combine age and CI quantile ranking into an age-network composite: ANC=*α*Age+(1−*α*) CI, with *α*=0.5, a remarkable correlation (*R*^2^=0.99, Fig. 3c) is achieved. By combining network information with age, the probability to identify individuals with a high credit limit reaches ∼70% at the highest earner level. Such a level of accuracy renders the model practical to infer individuals' financial fitness using network CI as we show next.

### Validation by marketing campaign

**Figure 4:**
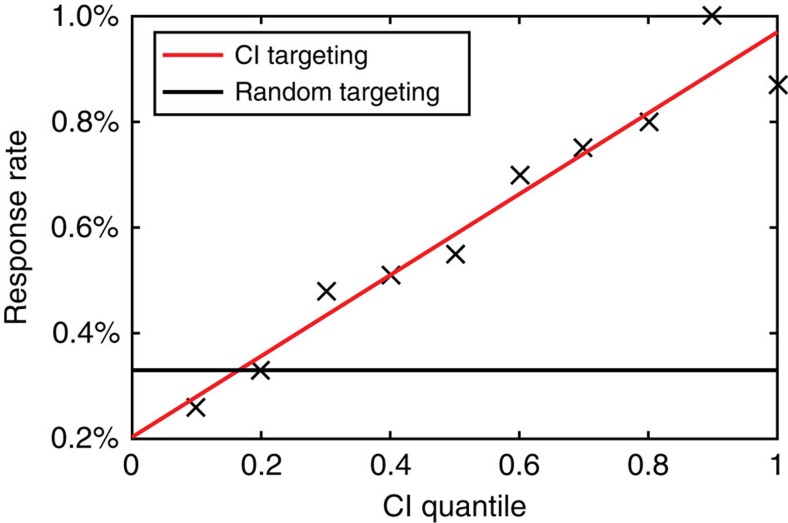
Response rate versus CI quantile in the real-life CI-targeted marketing campaign. The response rate increases approximately linearly with CI ranking. The CI-targeted campaign shows a threefold gain for the top influencers with high CI, as compared with a campaign targeting a randomized control group.

To validate our strategy, we perform a social marketing campaign whose objective is the acquisition of new credit card clients, by sending messages to affluent individuals (as identified by their CI values) and inviting the recipients to initiate a product request (Supplementary Note 8). We note that in this experiment we use an independent data set from a different time frame, and we use only the CI values extracted from the network to classify the targeted people. Specifically, we use the communications network resulting from the aggregation of calls and SMS exchanged between users over a period of 91 days. The resulting social network contains 7.19 × 10^7^ people and 3.51 × 10^8^ links. The campaign was conducted on a total of 656,944 people who were targeted by an SMS message offering the product according to their CI values in the social network. We also sent messages to a control group of 48,000 people, chosen randomly. To evaluate the campaign, we measured the response rate, that is, the number of recipients who requested the product divided by the number of targeted people, as a function of CI. In the control group, the response rate to the messages was 0.331%. Our results show that groups of increasing CI show an increase in their response rate, with a sound threefold gain in the rate of response of the top influencers (as identified by top CI values) when compared to the random case. When we compare the response of the high CI to the lowest CI people, the response rate increase fivefold. The results of the experiment are summarized in Table 1 and Fig. 4.

### Analysis of covariance

We note that our validation is indirect since it is not a direct prediction of financial status, but a rate of successful response to a marketing campaign. This success rate may actually depend on a number of other factors that may correlate with the network centrality. Thus, the CI metric may not necessarily be the only cause of the success rate of the targeted campaign (for instance, geographical location may be also important). To address this point, we perform an analysis of covariance^35^ on all of the features to which we have access (age, gender and registered zip code) to test the variance caused by the network metrics and other factors (details in Supplementary Note 5 and Supplementary Table 3). Analysis of covariance shows that the effects of the network metrics are independent from those of the other factors. The correlation between the CI and the fraction of wealthy people is positive and significant (*P*<0.001) in all groups of geographical communities, across genders, and among all age groups older than 24 years (Supplementary Fig. 6). The same significant results are also obtained under different thresholds of wealth. Such significant and robust network effects imply that network metrics may be a potential indicator for financial status.

### Network diversity and financial status

Our combined data sets also offer the possibility to test the importance of the diversity of links, as measured by ties to distant communities in the network not directly connected to an individual's own community, at the level of single individuals^4^,^5^,^6^. To this end, we first detect the communities in the social network by applying fast fold modularity detection algorithms (Supplementary Note 7; Supplementary Fig. 11)^36^,^37^. The diversity of an individual's links can be quantified through the diversity ratio DR=*W*_out_/*W*_in_ (ref. 10), defined as the ratio of total communication events with people outside their own community, *W*_out_, to those inside their own community, *W*_in_. This ratio is weakly correlated to CI (*R*=0.4), suggesting that it captures a different feature of network influence. We implement the same statistics of composite ranking as before, resulting in an age-diversity composite ADC=*α*Age+(1−*α*) DR, with weight *α*=0.5. The result (Fig. 3d) shows that ADC correlates with individual financial well-being, generalizing the aggregated results in ref. 6 to the individual level. Thus, the social metrics considered, DR and CI, express the fact that higher economic levels are correlated with the abilities to communicate with individuals outside one's local tightly-knit social community, a measure of Granovetter's ‘strength of weak ties' principle^4^ and to position oneself at particular network locations of high CI that are optimal for information spreading and structural stability of the social network. We note that no causal inference can be established with the present data.

## Discussion

This result highlights the possibility of predicting both financial status and benefits of socially targeted policies based on network metrics, leading to tangible improvements in social marketing campaigns. The high performance of CI among network metrics also suggests the possible role of accessing and mediating information in financial opportunity and well-being^5^. This has an immediate impact in designing optimal marketing campaigns by identifying the affluent targets based on their influential position in a social network. This finding may be also raised to the level of a principle, which would explain the emergence of the phenomenon of CI itself as the result of the optimization of socioeconomic interactions.

## Methods

### Code availability

Source code of the CI algorithm is available at the website http://www-levich.engr.ccny.cuny.edu/webpage/hmakse/software-and-data/. Other source code is available on request to the authors.

### Data availability

The data sets generated during and/or analysed during the current study are not publicly available for privacy reasons, but are available from the corresponding author on reasonable request.

## Additional information

**How to cite this article:** Luo, S. *et al*. Inferring personal economic status from social network location. *Nat. Commun.* **8,** 15227 doi: 10.1038/ncomms15227 (2017).

**Publisher's note**: Springer Nature remains neutral with regard to jurisdictional claims in published maps and institutional affiliations.

## Supplementary Material

Supplementary Information Supplementary Figures, Supplementary Tables, Supplementary Notes and Supplementary References

## Figures and Tables

**Table 1:** Results of the real-life marketing campaign.

**CI range**	**Count**	**Quantile**	**Answered yes**	**Response rate**
(0, 48)	66,495	0.1	170	0.26%
(48, 246)	65,164	0.2	218	0.33%
(246, 600)	65,961	0.3	316	0.48%
(600, 1,144)	65,376	0.4	332	0.51%
(1,144, 1,992)	65,477	0.5	363	0.55%
(1,992, 3,408)	65,477	0.6	458	0.70%
(3,408, 6,032)	65,736	0.7	493	0.75%
(6,032, 11,772)	65,641	0.8	555	0.8%
(11,772, 28,740)	65,683	0.9	657	1.0%
(28,740, 2,719,354)	65,683	1.0	573	0.87%

Individuals (‘Count') were targeted according to their quantile CI ranking in the whole social network obtained from phone communications activity. The response to the campaign (‘Answered yes') was computed to calculate the Response rate.
