# Supplementary material for: Inferring personal economic status from social network location
Source: Nat Commun. 2017 May 16;8:15227. doi: 10.1038/ncomms15227 (PMC5440802; doi:10.1038/ncomms15227)
Supplement: Supplementary Information — Supplementary Figures, Supplementary Tables, Supplementary Notes and Supplementary References [file ncomms15227-s1.pdf]

SUPPLEMENTARY INFORMATION

Supplementary Note 1 - Datasets

The present framework and data acquisition have gone through an extensive process of revision and approval that took more than one year and have IRB approval Protocol No. 2016-1418 at City University of New York.

In the framework of the study, the private and/or sensitive information of the telecommunications company clients was protected. In particular, the Bank didn't gain access to any individual information about the telecommunications company users. Similarly, the private information of the Bank's clients was protected in the framework of the study. In particular, the telecommunications company didn't have access to the individual information of the Bank's clients. The variables shared were revised to guarantee that the privacy of clients was protected.

All of our datasets are encrypted and securely stored. The mobile dataset consists of records of phone calls and SMS (short message service) metadata which was collected from clients of a major operator of a Latin American country. The dataset is anonymized. All the data are encrypted and stored in a server secured by enterprise-grade firewall. The records cover a period of 122 consecutive days. Each phone number was encrypted by a high level of hashing in order to eliminate all possible access to personal information. For our purposes, each CDR (Call Detail Record) is represented as a tuple ⟨x, y, t, dur, d, l⟩, where x and y are the encrypted phone numbers of the caller and the callee, t is the date and time of the call, dur is the duration of the call, d is the direction of the call (incoming or outgoing, with respect to the mobile operator client), and l is the location of the tower that routed the communication. Similarly, each SMS metadata record is represented as a tuple ⟨x, y, t, d, l⟩. We constructed a social network $G = (N, E)$ based on the phone call and SMS traffic. Both reciprocal and non-reciprocal links are preserved for further processing.

In inferring the real social network from the mobile network, we take the assumption that the communication demands are rigid against the cost, which is usually affordable to most families (∼USD $17 monthly cell phone service fee vs. ∼USD $600 monthly income in the year data was collected, respectively). Thus, the direct impact of an individual's financial status on the communication structure evidenced in the mobile phone network might be

limited. However, the financial cost of using phone services makes it possible that there is a systematic bias in how much wealthy individuals use the phone services relative to people that have less money to spend on phone calls. At this point, with the present data, we cannot rule out this possibility.

The financial dataset from a major bank in the same country was collected during the same time period as the mobile dataset. These data record financial details of 1.23×10^6 clients assigned unique anonymized identifiers over the same three-month period as the mobile network. The dataset consists of records of the bank clients' age, gender, credit score, total transaction amount during each billing period, credit limit of each credit card, balance of cards (including debit and credit), zip code of billing address, and encrypted registered phone number. A subset of 5.02×10^5 clients have an encrypted mobile phone number, thus enabling them to be matched with the mobile communication dataset. The phone numbers are encrypted in the same way as in the mobile dataset, which guarantees that the two datasets are matched. Excluding the information on credit lines, all other personal information is erased. We sum up the credit limits of all the credit cards of each account owner to represent the total credit limit of each individual.

In the absence of direct access to an individual's income and total assets, evaluating an individual's financial status remains an open question. In this dataset, we can access the following factors:

Transaction amount, which also directly reflects the individuals' consumption patterns. However, since it is common that one holds multiple accounts in different banks, and some of these may not be used at all, records in only one bank might not correctly reflect the real spending ability of an individual. Similar reasoning can be applied to total credit card balance per month, which could also lose its ability to measure one's financial status.

Credit scores assigned to individuals by credit scoring agencies are also good indicators of financial status. However, the values of credit scores are quite limited, ranging from 300 to 850. This limited range makes the credit score a low-resolution indicator of wealth that does not allow us to correctly classify a large number of people into well-defined financial classes. On the other hand, the credit limit ranges over three orders of magnitude, allowing us to correctly classify the entire population. Considering the weaknesses of the other features, total credit limit is the most convenient measure of personal financial status in the present dataset.

Instead of transaction amounts and credit scores, we choose the total credit limit which is assigned by the bank after comprehensive evaluation of an individual's financial status, as a proxy for financial status. Since detailed information on how the credit limit is assigned is not provided, there are several possible factors that could cause bias in inferring an individual's real economic status. These include the delay of credit limit in reflecting a change in an individual's financial status, possible correlation with the age of the account, and so on. In fact, the credit limit might be capturing the amount of information the bank has about the customer, instead of his/her actual income.

Supplementary Note 2 - Removing non-human-operated lines

Inferring social network structure through mobile phone data requires the removal of lines operated by non-humans. Due to privacy restrictions, we could not filter business landlines and spawn spreaders at the outset. Several ways of filtering the landlines were applied in previous works, including setting a cut-off threshold degree [1] or only considering reciprocal phone calls [2]. However, these methods usually also cut off some important human communication behavior in that particular window of observation. All communication events should be considered in evaluating the social network. Therefore, the key problem is to find a method to distinguish human- and non-human-operated lines while retaining maximal information about individuals' communication patterns.

Although we do not have the human/non-human label for the totality of the phone lines, which could separate at the outset the non-human-operated lines, we are in possession of the set of phone numbers registered with the bank dataset. These human-operated lines provide the possibility of supervising a machine learning process to learn the human behavior that separates them from robots and non-human-operated lines. We set up a hypothesis test by modeling the human-operated lines based on several variables. We first cluster the human-operated lines in a hyperspace. A new unlabeled node will be assigned a p-value according to its distance to the cluster. By carefully choosing a threshold of the p-values, we can label the node according to whether we accept or reject the hypothesis that the line is operated for personal use.

A training set consisting of the phone lines in the bank database (1.23×10^6 nodes), which is around 1% of all of the data in the entire network (1.10×10^8 nodes), was set up. We

define a call or message from phone number i to j as a 'communication event,' and denote the total number of communication events on the link as $W_{i\to j}$. The key assumptions of the model are the following:

1. Communication between lines of personal use is usually (but not always) reciprocal. This means that the fraction of paired communication events on human-operated lines is generally higher than that of unpaired ones. Namely, it suggests that although communication load difference D_i on every line:

$$D_i = \left| \sum_{j\in\partial i} W_{i\to j} - \sum_{j\in\partial i} W_{j\to i} \right| \tag{1}$$

should increase with degree k, it should be bound by an upper limit in the case of human-operated lines. Numbers operated for non-personal use like business hubs and spawn spreaders may have very large D_i because they are usually operated only for sending or receiving phone calls independently, but not for both at the same time.

2. Other types of business hubs may have large numbers of paired communications despite their limited D_i. These business hubs include the phone numbers for company landlines, roadside assistance, or other services requiring instant follow-up by the recipient of the phone call. To filter out these hubs we assume that the paired communication:

$$R_i = \sum_{j\in\partial i} \min(W_{i\to j}, W_{j\to i}) \tag{2}$$

also increases with k, but is limited for lines for personal use. The decay of the tail is supposed to follow a power-law due to the preferential attachment rule [2].

The last assumption is: 3. Most phone numbers in the network are for personal use, which results in the number of non-human-operated lines being small.

After we introduce these basic assumptions, empirical analysis can be applied to build a model describing human-operated line behavior. The model simplifies to a parametric probability distribution depending on two random variables D_i and R_i, and a variable maximum degree k which controls the parameters. Under the preferential attachment rule of assumption 2, it is reasonable to assume the distributions of both D_i and R_i for a given k deviate from a maximum entropy distribution and show a power-law tail. A good approximation is the log-logistic distribution:

$$P(D_i|k) \sim LL(d_i, \alpha_{\mathrm{D}}(k), \beta_{\mathrm{D}}(k)), \tag{3}$$

and

$$P(R_i|k) \sim LL(r_i, \alpha_{\mathrm{R}}(k), \beta_{\mathrm{R}}(k)), \tag{4}$$

where

$$LL(x, \alpha(k), \beta(k)) = \frac{(\beta/\alpha)(x/\alpha)^{\beta-1}}{[1+(x/\alpha)^{\beta}]^2}. \tag{5}$$

This also suggests the logarithm of both metrics follows a normal-like but exponential tailed logistic distribution:

$$P(\log D_i|k) \sim L(d_i, \mu_{\mathrm{D}}(k), s_{\mathrm{D}}(k)), \tag{6}$$

and

$$P(\log R_i|k) \sim L(r_i, \mu_{\mathrm{R}}(k), s_{\mathrm{R}}(k)), \tag{7}$$

where

$$L(x, \mu(k), s(k)) = \frac{1}{4s(k)} \mathrm{sech}^2\left(\frac{x-\mu(k)}{2s(k)}\right), \tag{8}$$

with $\mu(k) = \log(\alpha(k))$, and $s(k) = \frac{1}{\beta(k)}$. Based on the knowledge we have, this distribution is the best choice even though we cannot precisely provide an exact fitting. However, the fitting results strongly support the approximation geometrically (Supplementary Figure 1). The model involves four parameter sequences: $\hat{\mu}_{\mathrm{D}}(k)$, $\hat{s}_{\mathrm{D}}(k)$ and $\hat{\mu}_{\mathrm{R}}(k)$, $\hat{s}_{\mathrm{R}}(k)$. To determine the function of dependency, we pick the interval $k = 40$ to 160. We consider this a normal range of degrees wherein the nodes are almost all human-operated to fit the trend of μ and s. Adequate numbers of observers in each degree division guarantee the reliability of the results. The estimated $\hat{\mu}_{\mathrm{D}}(k)$, $\hat{s}_{\mathrm{D}}(k)$ and $\hat{\mu}_{\mathrm{R}}(k)$, $\hat{s}_{\mathrm{R}}(k)$ can be simply described by linear models within this range (Supplementary Figure 2, $R^2 > 0.98$). The relations are then used to predict parameters under other degree ranges.

After validating the assumptions, we are able to implement the learning process by performing a hypothesis test:

1. Fit the model of training data and get the sequence of estimated $\hat{\mu}_{\mathrm{D}}(k)$, $\hat{s}_{\mathrm{D}}(k)$, $\hat{\mu}_{\mathrm{R}}(k)$, and $\hat{s}_{\mathrm{R}}(k)$.

2. For each node i with given difference d_i, number of communication pairs r_i and degree k_i, calculate the p-value of $p_{\mathrm{D}}(i) = P(D < d_i|k_i)$, and $p_{\mathrm{R}}(i) = P(R < r_i|k_i)$.

3. Set a threshold p using the following test to classify the nodes:

If:

$$p < p_{\mathrm{D}}(i) < 1-p \quad \wedge \quad p < p_{\mathrm{R}}(i) < 1-p \tag{9}$$

then i is a human-operated line. Otherwise a p-value outside the range defined above will be rejected by the null hypothesis: $H_0 \to i$ is a human-operated line. It will be labeled as a non-human-operated business hub due to its extraordinarily unbalanced communication pattern or large volume of communication events.

Last but not least, the threshold p should be optimized. Suppose the network follows the exact distribution given by the model above. The fraction of outliers (non-human-operated lines) ϵ is exactly $2p$. The difference $\epsilon - 2p$ can be approximately regarded as the number of non-human-operated lines or 'outliers'. Supplementary Figure 3 is the plot of p over $\epsilon - 2p$. A maximum is reached when $p \sim 1.6 \times 10^{-5}$. At that point, the filter is the most sensitive to detecting outliers since it covers the boundary of human- and non-human-operated nodes.

The result of data filtering is shown in Supplementary Figure 4. The final network has 1.07×10^8 nodes (97.27% of the total data) and 2.46×10^8 links. There are 4.51×10^7 reciprocal social ties. The size of the giant connected component is 99.2% and the average degree is 4.7. The maximum degree k is 1056 and the maximum total communication load of a single node is $\sim 10K$ including messages and calls, which is reasonable for a person who is active in business contacts during a three-month period.

Supplementary Note 3 - Entropy Analysis

In order to explore the structural differences between people with different levels of credit limits, we performed an entropy analysis. First, we choose people within the top 5% and bottom 5 to 10% credit limit percentiles, representative of the wealthy and poor populations respectively. Then, we randomly divided both groups into 20 small subgroups where each subgroup contained $N(0) \sim 2700$ bank clients. Next, we expanded each subgroup's contacts by a distance ℓ to get a subnetwork and clustered the nodes in the subnetwork through modularity analysis (Supplementary Note 6) into different communities, finally counting the number of nodes inside each community (n_i). The entropy of this subnetwork is defined as:

$$S = -\sum_i p_i \log p_i, \tag{10}$$

where $p_i = \frac{n_i}{\sum_i n_i}$ is the fractional size of community i. Also, we introduced two indicators: (1) $R_{\rm n}(\ell) = N(\ell)/N(0)$, which is the ratio between the size of the augmented network $N(\ell)$

and the size of the initial subgroup $N(0)$, and (2) $R_c(\ell) = C(\ell)/C(0)$, where $C(\ell)$ is the number of communities in the augmented network and $C(0)$ is the number of communities in the initial subgroup. Supplementary Table 1 shows the results of entropy S, $R_n(\ell)$ and $R_c(\ell)$ across an average of 20 subgroups, with uncertainties.

The entropy in subnetworks generated from the poor population is higher than in subnetworks generated from the wealthy population, while the numbers of both the total communities and nodes are smaller. This suggests that the sizes of the communities in the subnetwork of poor people are relatively more balanced than in the wealthy population. Namely, wealthy people are more likely to form larger and more closely-connected communities which result in relatively low entropy. The result of R_n and R_c shows the significant difference between the size and diversity of the subnetworks of the wealthy and poor populations. By expanding their contacts, people with higher credit limits 'collect' more people and more communities. Such differences exist even when we increase the value of ℓ to 4. The result of the entropy analysis implies that the network structure of these two groups may be significantly different. Wealthy people have higher diversity in mobile contacts and are centrally located, surrounded by other highly-connected people (network hubs).

Entropy analysis results also provide evidence of homophily, which implies that there exists a higher probability that two wealthy individuals are connected than that a wealthy individual and an extremely poor individual are connected. Since society is known to have this strong stratification property embedded in social networks, we would expect that this feature is expressed in our network. For example, if wealth implies higher degree, then homophily will lead to degree correlations, higher k-shell scores for wealthy individuals, and higher CI. Thus, part of the effect we observe in the present study might be due to the effects of homophily. However, the exact picture of how homophily affects the wealthy population is still to be discovered.

Supplementary Note 4 - Social Network Metrics

In order to capture the analytical evidence describing the effects shown in Figs. 1a–d, we introduce four different metrics to evaluate network influence [3, 4].

1. Degree centrality k_i is the simplest evaluation of an individual's local contact size. It requires minimum information and is easy to calculate. Other centralities such as be-

tweenness centrality cannot be efficiently calculated in our networks due to their nonlinear running times with system size.

2. k-core and k-shell index k_s [5] capture the centrality of a node in the global network by the method of k-shell decomposition. In this method, nodes are removed iteratively if their degree $k_i < k$ until all the remaining nodes have degree equal to or greater than k. These nodes remain in the k-core of index k. The largest k-core a node can hold is the k-shell index k_s, which means the node is in the 'shell' of the k'th core but outside the $k+1$'th core. The k-shell or k-core number is a global metric. It has been proven efficient in identifying single influencers through the SIR model [5]. The k-shell index requires the overall information of the network. It is a quantity that does not allow one to classify the nodes with high resolution: there usually exist a few k-shells in the whole system, each containing many of the nodes in the network. Fig. 1c is a schematic example of a k-shell in a network.

3. PageRank [6] is as eigenvalue centrality metric used to evaluate the probability that information or knowledge will likely visit a node through a random walk. PageRank is calculated through an iterative algorithm in which nodes collect PageRank values from their neighbors in every iteration. For simplicity, each node is initially assigned a value of $\mathrm{PR}(i) = 1$. During each iteration, node i collects a PageRank value through the link pointed from its neighbor j ($j \to i$) as the PageRank of an adjacent node divided by its outbound degree k^j_{out}. Namely,

$$\mathrm{PR(i)} = (1-d) + \sum_{j \in (\partial i \to i)} \frac{\mathrm{PR}(j)}{k^j_{\mathrm{out}}}. \qquad (11)$$

Here $\partial i \to i$ is the set of points which have outbound links to i, and d is a damping factor which we choose as 0.7 in our work. When a converging threshold (10^{-4}) is reached, the iteration stops and outputs the final result of PageRank.

Although PageRank was originally proposed for ranking websites, it has also been applied in social network analysis. Given the assumption that senders of messages or makers of phone calls are likely to be the ones providing the information being communicated, PageRank is a good metric to evaluate the likelihood that an individual captures the information spreading in the network. Similarly to k-shell, PageRank requires the global information of the whole network. However, it is easy to update when the network changes.

4. Collective Influence (CI) is an algorithm to identify the most influential nodes via optimal percolation [7]. Rather than the above heuristic metrics, Collective Influence is

introduced by a theoretical approximation of the solution to a problem of influence maximization in locally tree-like social networks [8]. CI minimizes the largest eigenvalue of a modified non-backtracking matrix of the network in order to find the minimal set of nodes to disintegrate the network. It has been shown that this process maximizes the spread of information via a threshold model of spreading and also provides the most important nodes for the integrity of the network (optimal percolation). Each node is associated with a CI value, and those with the top CI values are the most influential nodes in the network. The definition of CI is given by:

$$\mathrm{CI}(i) = (k_i - 1) \sum_{j \in \partial \mathrm{Ball}(i,\ell)} (k_j - 1), \tag{12}$$

where the $\mathrm{Ball}(i, \ell)$ is defined in the text. We should note that the mobile communications network is a typical small world network (average path length $< \ell >\sim 8.9$), and the radius ℓ of the ball is limited by the network diameter.

Of the metrics we investigated so far, CI draws our attention since in practice, it has advantages in resolution, correlation with wealth, and scalability to massively large social networks. On the “global versus local” issue, we point out that while CI comes from a global theory of maximization of influence, it represents a local approximation in a sphere of influence of finite radius ℓ. Thus, it is a convenient way to quantify influence in large social networks due to its scalability. Furthermore, in cases where the whole picture of global connectivity is incomplete, the local connectivity up to a few layers ℓ might be enough to define network influence and predict the financial status of an individual. On the other hand, we have shown that global quantities like the k-core are also good for capturing an individual’s financial status. Indeed, the global k-core contains nested structures of relatively large degrees, which somehow resemble the concentric spheres of influence of a high-CI node. However, the k-core suffers from resolution problems: wealthy people might be located preferentially in the core of the network, but this core is too large to locate them with accuracy. For instance, there are only 25 k-cores in the whole network (Fig. 2b) to separate one hundred million people, while CI has a larger resolution spanning eight orders of magnitude. Thus, in practical terms, CI presents advantages both in resolution and in high correlation with wealth.

Also, CI represents a balance between a global maximization of influence and its local approximation in successive layers, allowing one to use the CI metric in large-scale datasets

composed of hundreds of millions of individuals. Overall, we emphasize that CI is just a useful strategy for the reasons shown above, but by no means the only or best way to express the wealth of individuals. More generally, supervised machine learning can be applied to the problem of predicting an individual's credit score based on a number of features. These methods could include not only CI but also the other measures discussed, along with many other standard network metrics. Augmenting these measures for determining feature importance could allow us to better assess which features are important to determine the wealth of individuals with higher accuracy than that shown by CI in the present study. The prediction model will give standard measures of features' importance in further studies when we have access to more data. Future work will follow this promising direction.

Supplementary Note 5 - Financial parameters and other factors

We use the following statistics to identify economic effects: First, we separate the individuals into groups on sampling grids in variable space (1D as segment bins and 2D as grids). In each group (with more than 10 people for statistical significance), we count the fraction of wealthy individuals, defined as those individuals in the top 4-quantile $Q > 0.75$ or who have a total credit limit greater than USD \$4,000 (converted).

Besides the credit limit, transaction amount and credit score the bank data also provides the information of the clients' birth years. Age as a variable is independent from the network metrics (Supplementary Table 2) and correlates with the percentile-ranking credit limit ($r = 0.42$). However, we do not know the model used by the bank to assign the credit limit, so the age may be a complex reflection of the mixed effects of both increased income and increased account history. Thus, the correlation between age and credit limit might not be capturing only variation in actual wealth but also the amount of information the bank has about the customer.

To quantitatively evaluate the variance caused by network metrics when combined with other factors, we employed Analysis of Covariance (ANCOVA) [9]. ANCOVA is an analysis method which conducts regressions between covariate (CV) and dependent variables (DV) under different groups of categorical independent variables (IV). In this case, regression was made between covariate CI and the dependent variable, the fraction of wealth. As in Fig. 2d, CI is divided into 100 partitions. Based on the information to which we have

access, ANCOVA was applied separately among the following independent variables: gender, age, and residential communities. Gender was naturally divided into two groups. Age was grouped year by year from 18 to 65 in a total of 48 groups. The communities were identified by their registered zip code. To reduce the dimensionality of the problem and directly quantify the effect of geographical location, we first sorted the communities by the fraction of wealthy people inside and divided them into 50 balanced groups. We assigned to every community an 'Index of Community Wealth' (ICW), which is the quantile ranking of each group that the community belongs to.

The correlation between IVs and CV are shown in Supplementary Table 3. The negligible correlation between these variables ensures the basic assumption of independence in ANCOVA. Also, in order to test the robustness of our results, the same method was applied under different thresholds of credit limits to define the wealthy population: Q = 0.75 (the threshold we used), 0.85 and 0.95.

The basic output of ANCOVA is a series of p-values showing the significance level of the regression model between CV and DV in different IV groups, and the analysis of variance (ANOVA) [9] evaluating the significance of the IVs' effects. The estimated slopes with 95% confidence intervals are shown in Supplementary Figure 6. Our results show the following:

1. All IVs' effects are significant ($p < 0.001$); namely, the fraction of wealthy people is different among different groups of gender, age or communities.

2. Inside most groups of each IV, the variation caused by CI is also significant ($p < 0.001$). The only exception is that CI's effect is only significant when the clients are older than 24 years (Supplementary Figure 6b). This result indicates that the effect of network metrics, in most cases, is independent from the other known factors.

3. The slope of regression varies in different groups. However, all the slopes with significant values are positive.

4. The results of 1 to 3 above are robust under different thresholds of credit line, so Fig. 2 is also similar under different thresholds. Therefore, we focus our results on a given quantile threshold $Q = 0.75$ for the remainder of the study. Although the violation of homogeneity in 3 prevents us from making a direct comparison between variables, these results imply that CI significantly and independently affects the fraction of the wealthy population.

Supplementary Note 6 - Correlation between network metrics and financial status

To compare the value of the social metrics to the economic status of individuals, we have to draw out the best one to describe network location influence effects. We sum up all the age groups and consider the effect of network metrics to demonstrate the effects of each variable.

The reason for using the aggregated model instead of the direct correlations at the individual level is because the regression models at the individual level are based on certain assumptions that are not satisfied by our data. Thus, we were unable to apply regression models at the individual level, and instead provide data at an aggregated level. The failure of regression models at the individual level is due to two reasons:

1. The distribution of credit limit (CL) for a given level of ANC [which is a log-normal-like distribution with several peaks located at integers such as 50,000 or 100,000 (Supplementary Figure 7a)] is not invariant under changes in ANC. That is, the distribution changes shape when ANC increases, showing an increasing fraction of high-CL population while the fraction of people around the mean value stays unchanged (Supplementary Figures 7b–d). Such behavior directly violates the constant variance assumption of regression models and causes the data to be poorly captured by least-square regression models.

2. Besides the above fluctuations in the credit limit, other unknown factors may provide random fluctuations in inferring individuals' financial status. Such combined random effects are considerable at the individual level. However, aggregation models reduce the fluctuation caused by random factors, and the effect of the network emerges at the population level.

Thus, we adjust our statistical model to reflect the complexity of economic effects from network metrics and aggregate the data as follows:

First we separate the individuals into groups of sampling grids in a variable space (in 1D as segment bins and in 2D as grids). In each group (with more than 10 people for statistical significance), we count the fraction of wealthy individuals defined as those individuals in the top 4-quantile $Q > 0.75$ or who have a total credit limit greater than (equivalent to) USD $4,000. The dependence of our results on different wealth thresholds is provided in Supplementary Note 5.

Besides the degree, the volume of communication may have correlations with economic

status since we could not eliminate the systematic bias caused by phone call service fees. We investigate the correlation between the fraction of wealthy people and the average communication load per link: $\mathrm{AVL}_i = \frac{W_i}{k_i}$, where W_i is the volume of communication events and k_i is the degree of node i. The regression result shown in Supplementary Figure 9 shows that there is no significant correlation between the average communication volume per link and the fraction of wealthy individuals. Therefore, the effect of communication volume is negligible in comparison with the other variables considered in this study.

Supplementary Figure 8 shows the results. The large fluctuation in degree for higher quantiles in Supplementary Figure 8a implies that the effect of degree involves complex social patterns rather than only the local properties of the degree of the node. Thus, we abandon the use of degree for further study as an indicator. k-shell is good enough to present a positive correlation of high network location influence. However, due to the limited values of k-core, it cannot provide finer resolution for prediction (Supplementary Figure 8b). Therefore, k-shell is also not considered for further studies as an indicator. The performance of PageRank (Supplementary Figure 8c) with a slightly negative correlation suggests that it is not the optimal variable to rank economic status, and thus it is not considered herein.

Finally, CI (Supplementary Figure 8d) shows strong global correlation and satisfying resolution, which makes it a convenient metric for quantifying the influence of network location. The strong correlation with CI is invariant under different radii of influence ℓ (Supplementary Figure 10).

We notice a non-monotonic oscillatory behavior of the fraction of wealthy people when using k and CI as variates (Supplementary Figures 8a and 8d). This effect is complex and cannot be captured by either the degree or CI, and may not be limited to local properties. The oscillation is reduced when using CI in the analysis, and this is one of our reasons for choosing CI as a potential predictor. We will continue investigating the non-monotonic pattern in future work.

Supplementary Note 7 - Modularity and Diversity ratio

Additional research on modularity was implemented as follows. Personal structural hole [10] effects were evaluated by the ratio of total weights attached with nodes outside a community k_{out}, to those inside a community k_{in}. A fast community detection algorithm introduced

by Blondel *et al.* [11] was implemented in this work. The algorithm aims to maximize the modularity function [11, 12]:

$$Q_m = \frac{1}{W}\sum_{i,j}[W_{ij} - \frac{W_i W_j}{2m}]\delta(c_i, c_j), \tag{13}$$

where W_{ij} is the number of communication events loaded on link i, j and c_i is the community label of node i. $W_i = \sum_{j\in\partial i} W_{i,j}$ and $W = W_{ij}\sum_{i,j}$. The global maximization of modularity was achieved by iteratively calculating the local maximization of normalized networks based on communities. Different communities were labeled during each iteration. Among all the communities, we chose the clustering of the second iteration to control the average scale of the community to 10^2. There are 4.92×10^5 communities inside the network. The distribution of community sizes is fat-tailed with a largest community size of 10^6 (Supplementary Figure 11). The fraction of wealthy individuals inside each community is independent of the size of the community ($r < 0.05$).

After we label the network with its communities, we can evaluate an individual's structural hole effect [10] by introducing the diversity ratio DR. DR is defined by the ratio of total communication events with people outside one's own community W_{out} to those with people inside the community, namely W_{in}, DR $=W_{\text{out}}/W_{\text{in}}$. The ratio is weakly correlated with CI ($r = 0.4$). The same statistic of composite ranking was implemented as CI with the same number of statistic segments and composite factor $\alpha = 0.5$ as in the text. The result (Fig. 3d) shows that the structural hole effect also has a strong correlation with the distribution of affluent individuals while it is weakly dependent on CI. This result confirms the importance of the ability to communicate with outside communities via "weak ties" for personal economic development [13].

Supplementary Note 8 - Marketing Campaign

In the marketing campaign, clients were approached by SMS messages offering a benefit. In the text we sent during the campaign, we did not provide a specific product. Instead, the only information we provided was to notify the client that he/she was eligible for an offer from the bank. This somehow eliminated the bias caused by the nature of a product which may have a different appeal to wealthy or poor people. We sent the following messages:

Request your credit card with benefits from (Bank_name) by calling at (Bank_phone_number).

Fees and requirements at (Bank_url).

(Bank_name) has a special offer for you. If you're interested call at (Bank_phone_number). Fees and requirements at (Bank_url).

(Bank_name) has a credit card fit for you. Request it by calling at (Bank_phone_number). Fees and requirements at (Bank_url).

(Bank_name) has a credit card with benefits. Request it at (Bank_phone_number). Fees and requirements at (Bank_url).

(Bank_name) offers you a credit card with benefits. Request it by calling at (Bank_phone_number). Fees and requirements at (Bank_url).

(Bank_name) has an exclusive offer for you, call at (Bank_phone_number). Fees and requirements at (Bank_url).

Supplementary Table 1. Results of the group entropy analysis for the wealthy population (with quantile ranking $Q > 0.95$) and poor ($0.05 < Q < 0.1$) population.

		S	$R_c(\ell)$	$R_n(\ell)$
$\ell = 1$	wealthy	6.37±0.12	5.5±0.4	9.3±0.7
	poor	6.68±0.10	4.3±0.3	7.1±0.5
$\ell = 2$	wealthy	7.94±0.10	141.3±4.7	$6.3 \pm 0.2 \times 10^2$
	poor	8.38±0.14	101.6±3.4	$3.1 \pm 0.1 \times 10^2$
$\ell = 3$	wealthy	9.11±0.11	443.0±11.5	$7.6 \pm 0.4 \times 10^3$
	poor	9.30±0.12	390.9±6.0	$4.9 \pm 0.4 \times 10^3$
$\ell = 4$	wealthy	10.23±0.02	565.4±10.7	$5.10 \pm 0.04 \times 10^4$
	poor	10.23±0.04	517.0±9.0	$4.23 \pm 0.05 \times 10^4$

Supplementary Table 2. Correlation (r-values) between the metric centralities obtained from the social network and age.

	k	k-shell	PageRank	$\log_{10}$CI
Age	-0.021	-0.016	-0.033	-0.007
k		0.972	0.648	0.953
k-shell			0.589	0.960
PageRank				0.575

Supplementary Table 3. **Correlation between covariate CI and independent variables: age, gender and Index of Community Wealth (ICW).** The correlation between gender and other features is presented through the Point-Biserial correlation coefficient, and other correlations are Pearson correlations. Point-Biserial correlation coefficients quantify the male as 1 and female as 0 and are defined as: $r = \frac{\bar{X_1}-\bar{X_0}}{s_{n-1}}\sqrt{\frac{n_1 n_0}{n(n-1)}}$. n is the total number of samples. n_1 and n_0 refer to the population inside each group. $\bar{X_1}$ and $\bar{X_0}$ are the means of the variables in each group. s_{n-1} is the estimated unbiased standard deviation of X: $s_{n-1} = \sqrt{\frac{1}{n-1}\sum_{i=1}^{n}(X_i - \bar{X})^2}$.

	CI	Gender	ICW
Gender	-0.0419		
ICW	-0.0093	0.0131	
Age	-0.0007	-0.0116	-0.0022

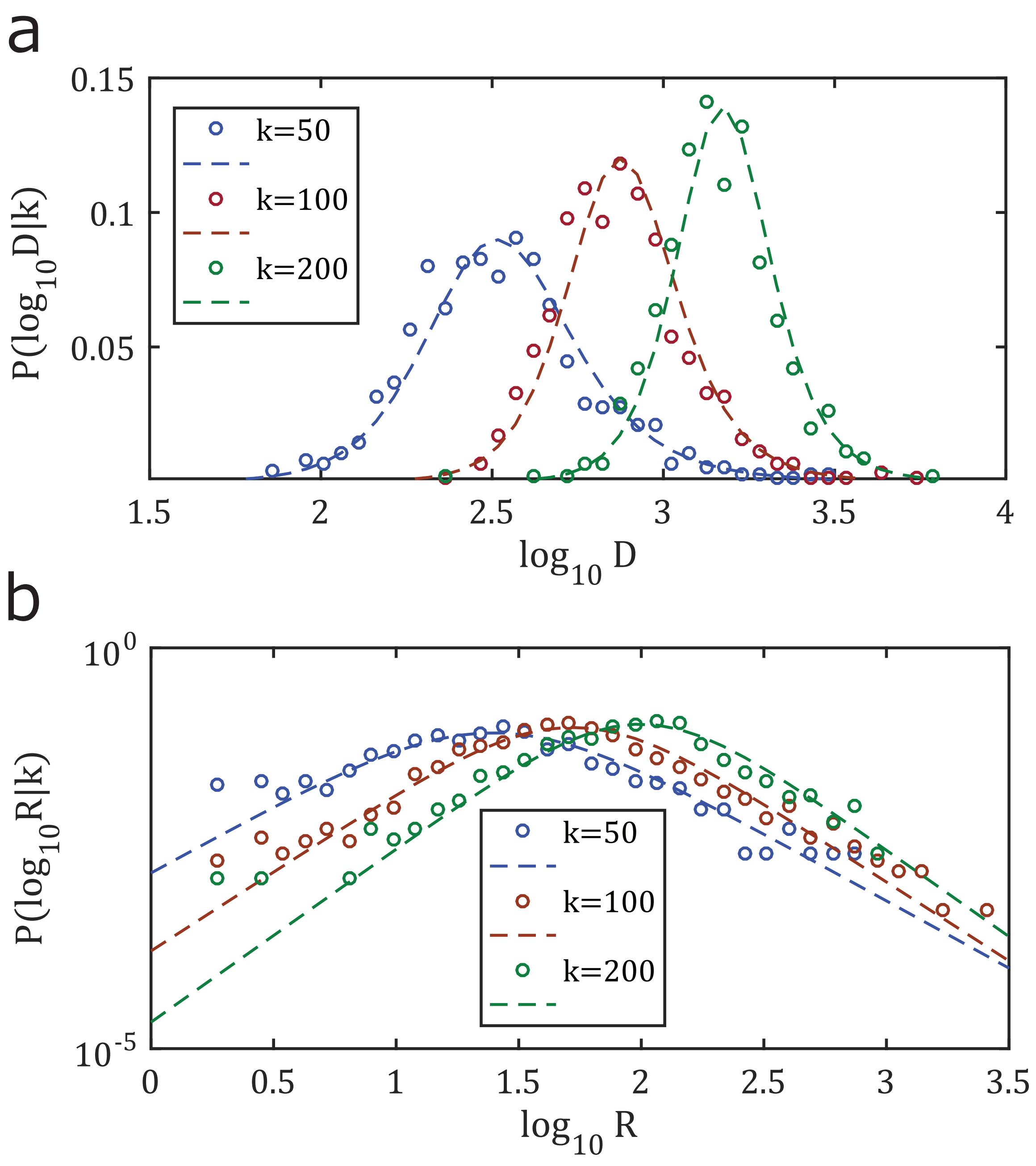

Supplementary Figure 1. **Logistic fitting result for $k = 50$, 100 and 200.** The result of paired communication R is presented in log-log scale in order to highlight the fitting for the exponential tails.

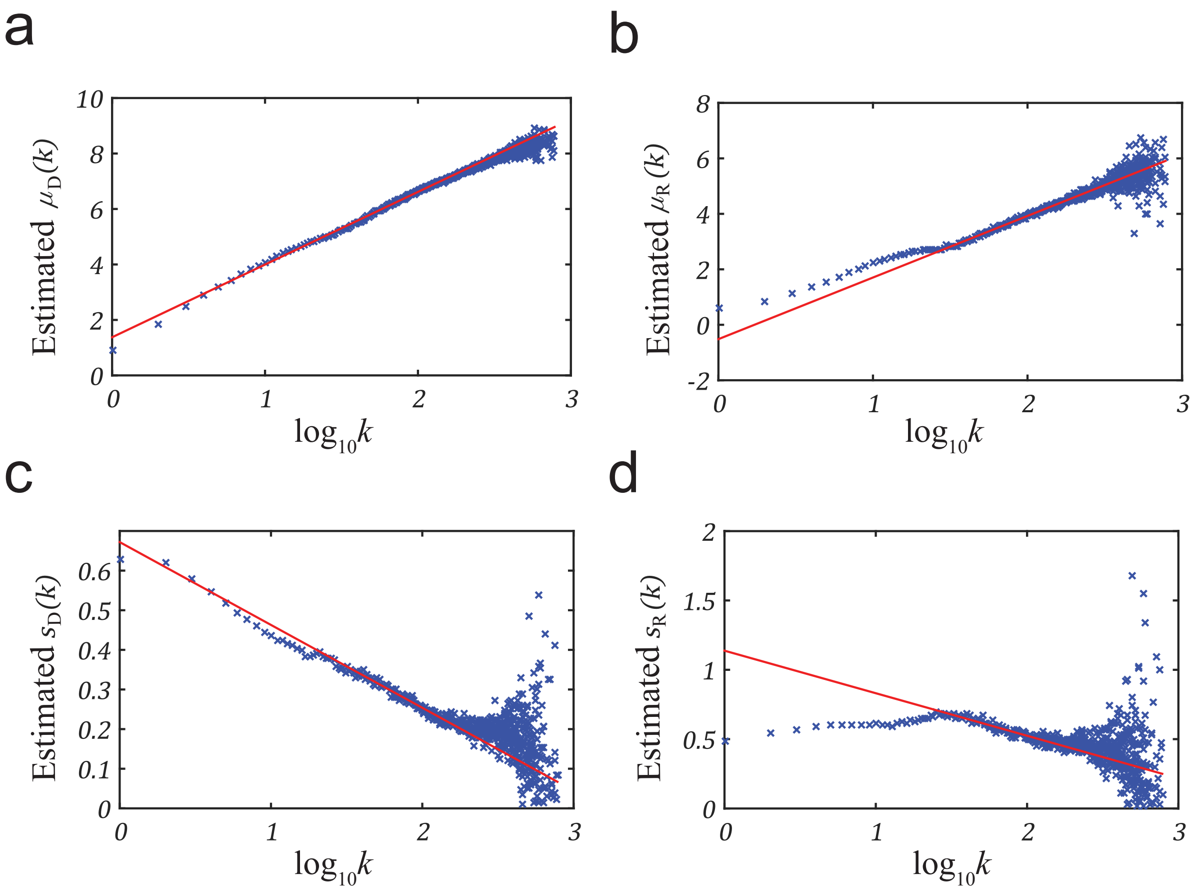

Supplementary Figure 2. **Scaled parameter estimation and its linear fitting:** (a) $\hat{\mu}_{\mathrm{D}}(k)$, (b) $\hat{s}_{\mathrm{D}}(k)$, (c) $\hat{\mu}_{\mathrm{R}}(k)$, (d) $\hat{s}_{\mathrm{R}}(k)$.

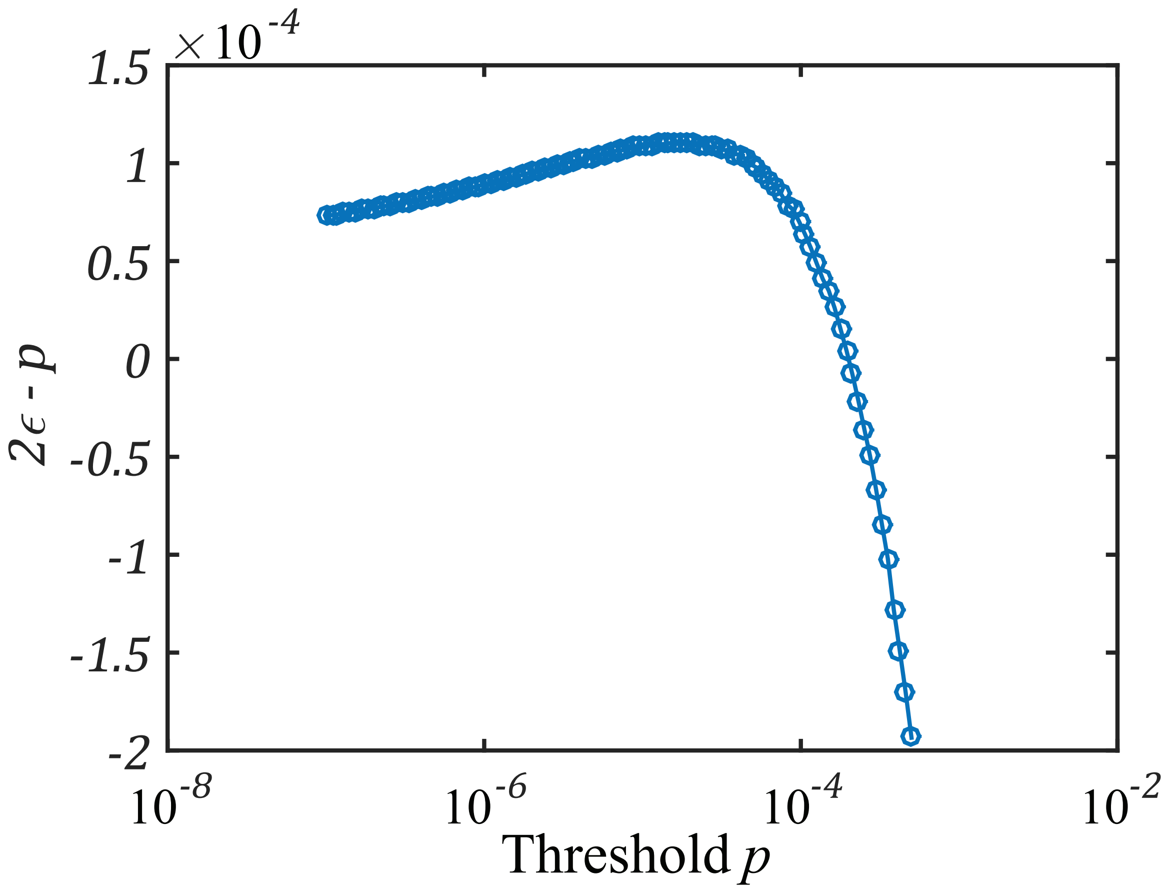

Supplementary Figure 3. **Number of outliers $\epsilon - 2p$ vs cut-off threshold p.** Maximum is reached when $p \sim 1.6 \times 10^{-5}$.

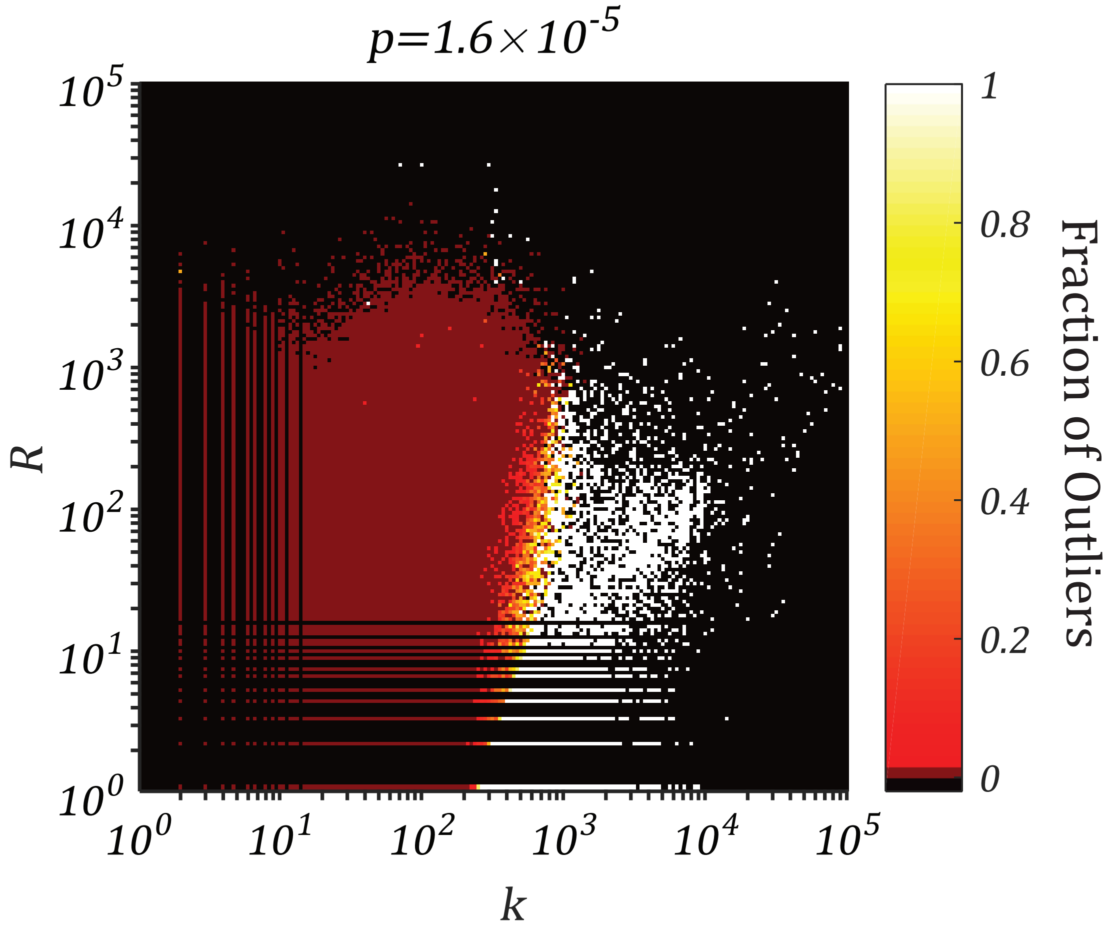

Supplementary Figure 4. **Final result of data filtering.** The result is presented in the space of k and communication pairs R. The data points were put into a grid bin of 200×200. The color represents the fraction of outliers in each bin. The filter gives us a gradual boundary of human- and non-human-operated lines.

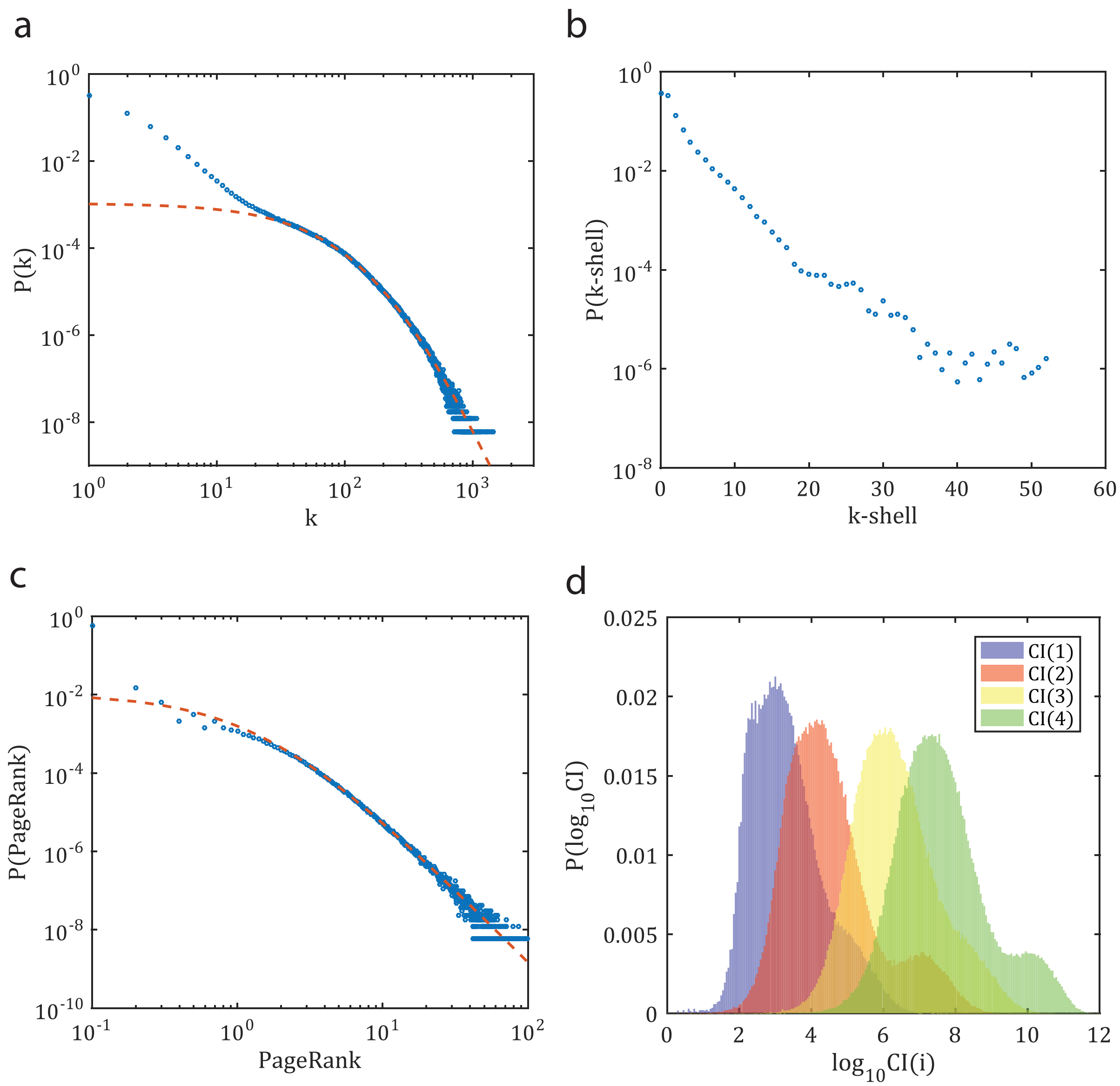

Supplementary Figure 5. **Distribution of network metrics.** **(a)** degree, **(b)** k-core, **(c)** PageRank, and **(d)** Collective Influence (ℓ =1 to 4). Collective Influence follows a double-tailed distribution. A small peak for larger CI emerges for even ℓ.

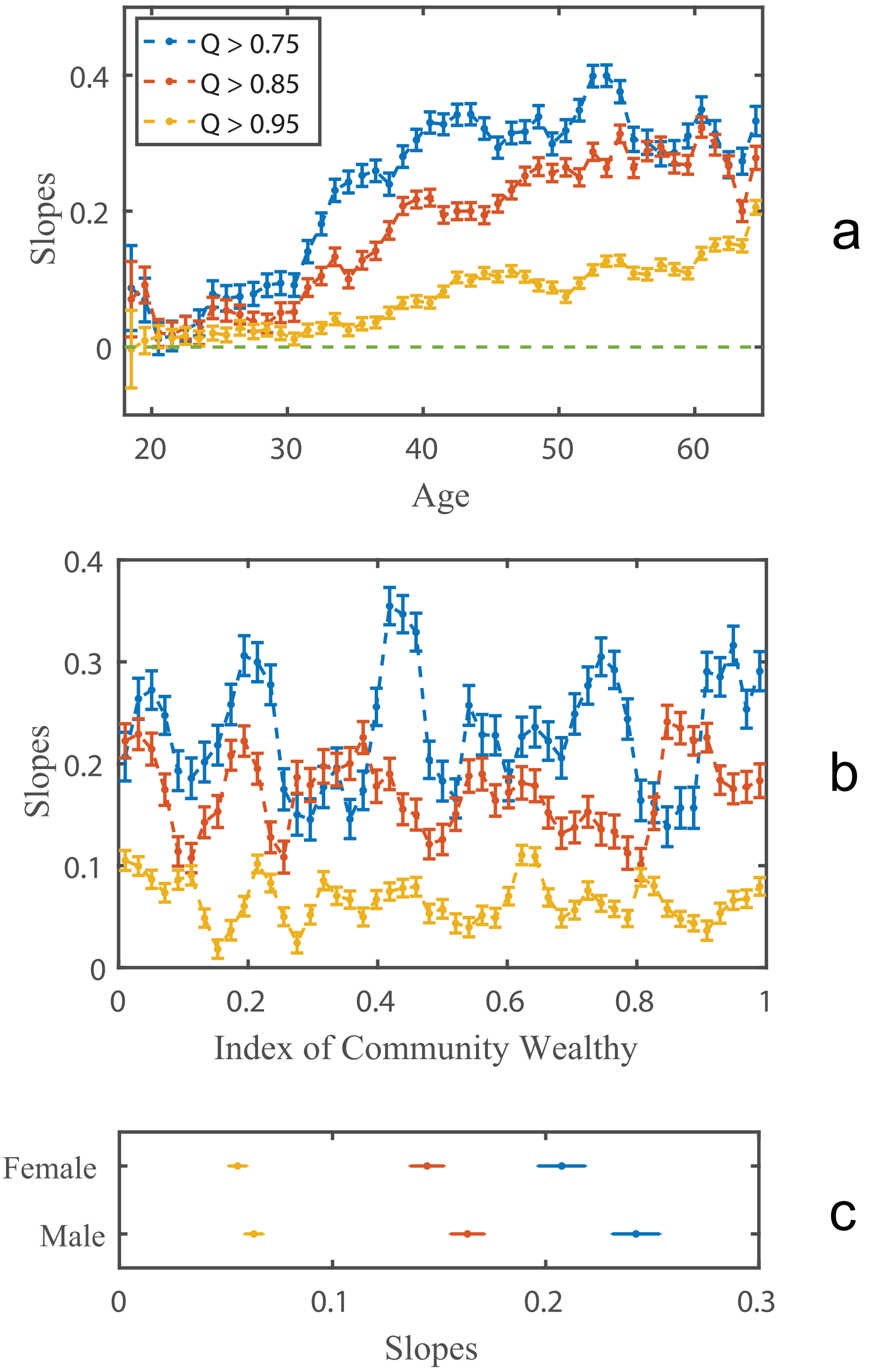

Supplementary Figure 6. **Estimated slopes in different groups of independent variables.** (a), Age, (b), Index of Community Wealth (ICW), and (c), Gender. 95% confidence interval is marked by error bars in the plot. Different thresholds of wealth Q are labeled by different colors.

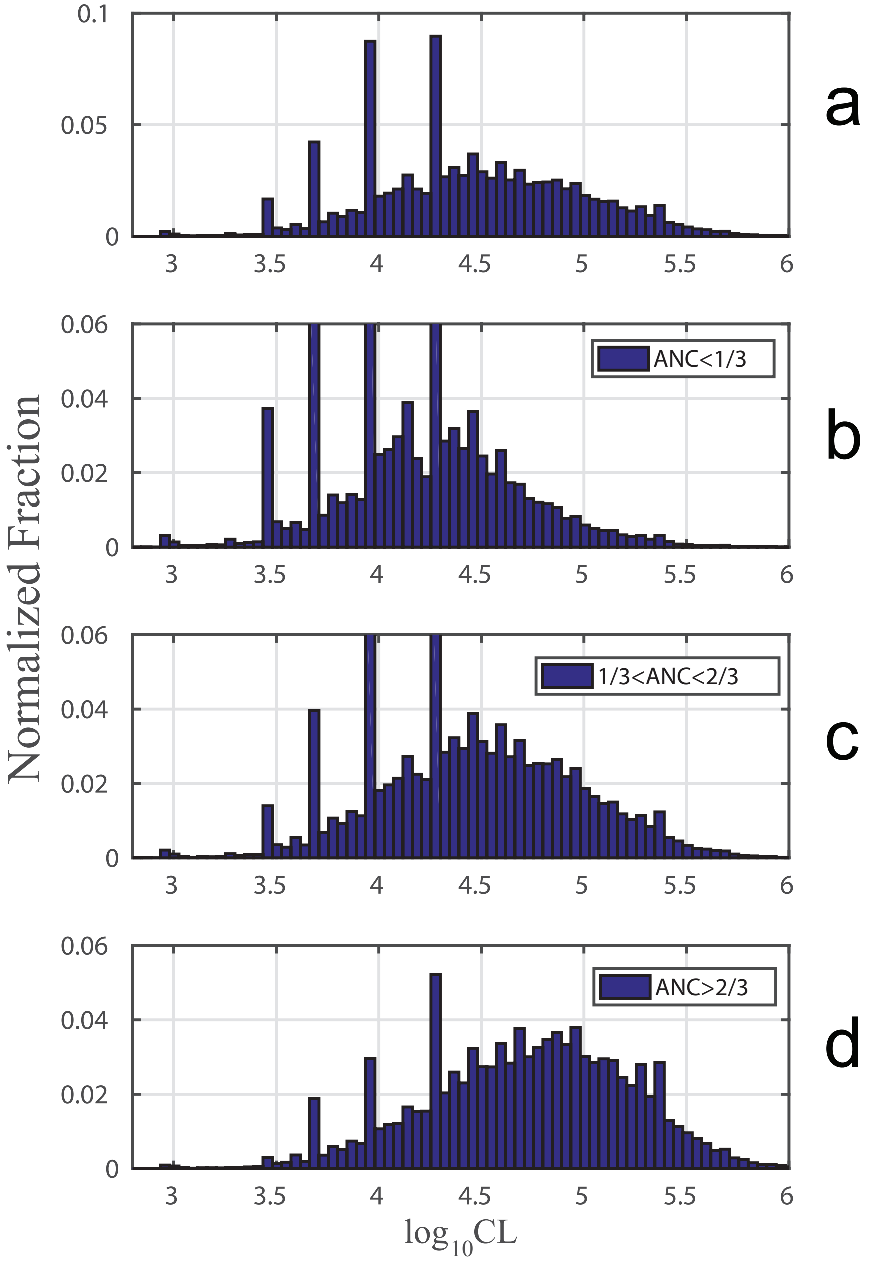

Supplementary Figure 7. **Distribution of Credit Limit (CL) under different age-network composite (ANC) groups.** The distribution is not invariant under changes in ANC.

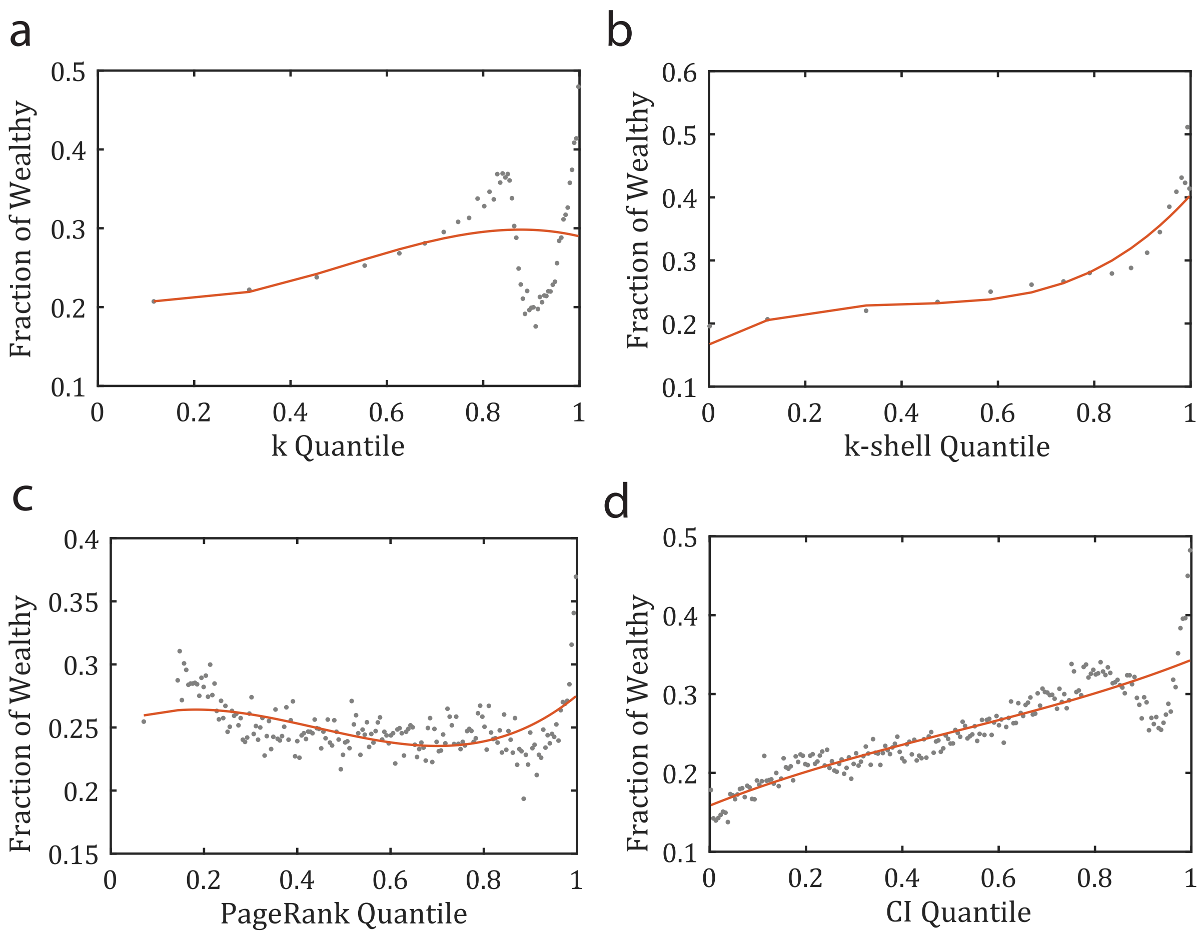

Supplementary Figure 8. **Fitting results of wealthy population vs. network influence metrics along with corresponding R^2 values.** **(a)** Degree (0.51), **(b)** k-core (0.99), **(c)** PageRank (0.28), and **(d)** Collective Influence (0.80). All variables are normalized to [0, 1] by the quantile ranking to ensure an adequate number of data points in each partition. The entire quantile ranking is divided into 200 segments from minimum to maximum. Only those groups with population larger than 10 are shown on the plot. Out of the four metrics, CI is the most convenient for capturing high correlations and presenting a large range of values that allow us to classify the whole population.

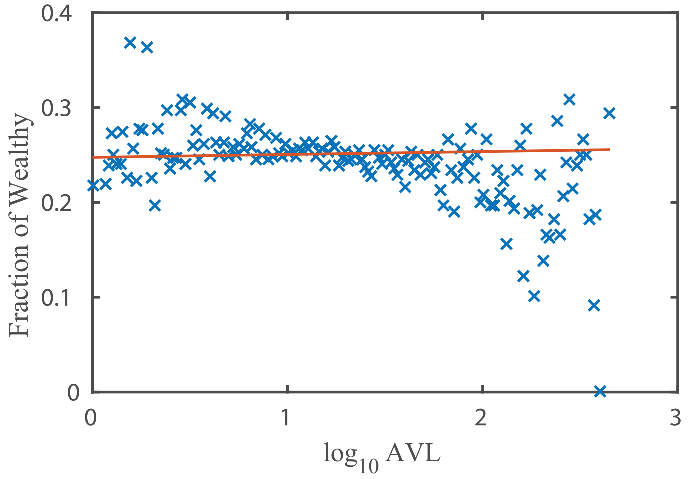

Supplementary Figure 9. **Fraction of wealthy people vs. average communication event load per link (AVL).** AVL is in log-10 scale and divided into 200 partitions. Each group with a population of more than 10 is considered in counting the fraction of wealthy people inside the group.

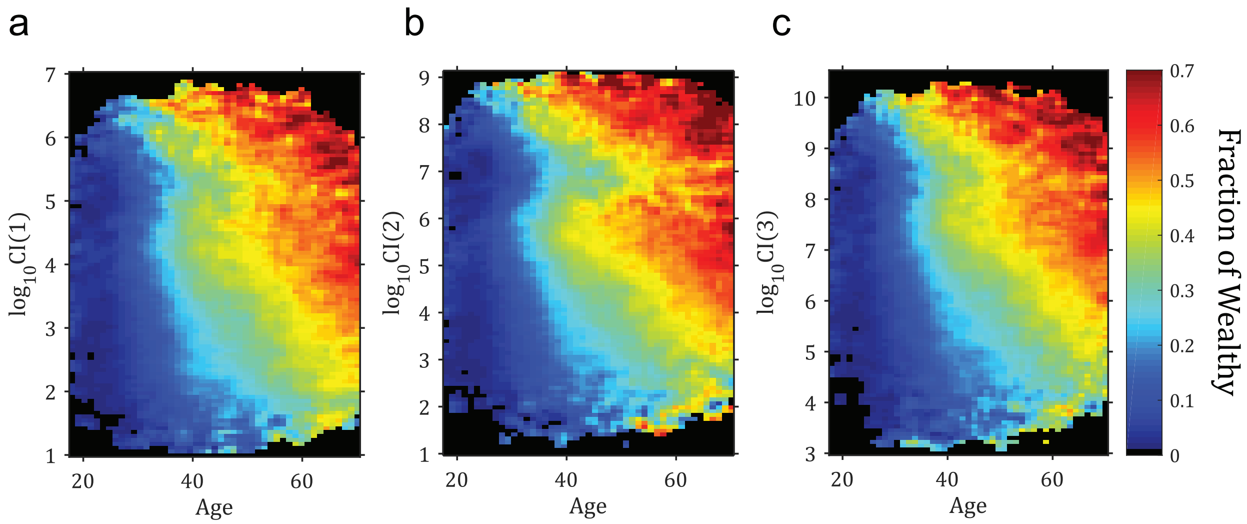

Supplementary Figure 10. **Fraction of wealthy people in each group against age and logarithm collective influence for different radius.** Radii ℓ range from 1 to 3. Communities are determined by 200 segments covering from the bottom 1% to top 1% of CI values. Only those groups with population larger than 10 are shown on the plot.

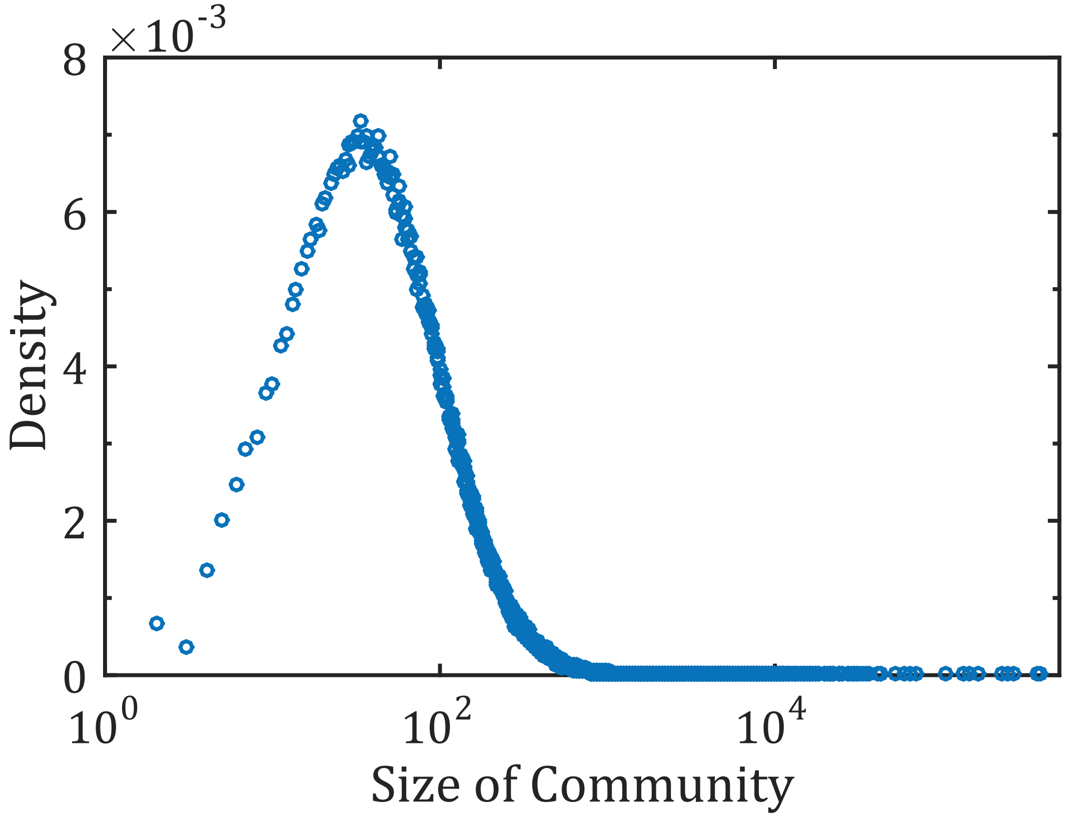

Supplementary Figure 11. **Distribution of community sizes in the entire social network at second iteration.**

Supplementary References

---

[1] Eagle, N., Macy, M. & Claxton, R. Network diversity and economic development. *Science* **328**, 1029–1031 (2010).

[2] Onnela, J.-P. *et al.* Structure and tie strengths in mobile communication networks. *Proc. Nat. Acad. of Sci.* **104**, 7332–7336 (2007).

[3] Wasserman, S. & Faust, K. *Social network analysis: Methods and applications*, vol. 8 (Cambridge University Press, Cambridge, UK, 1994).

[4] Freeman, L. C. Centrality in social networks conceptual clarification. *Soc. Networks* **1**, 215–239 (1978).

[5] Kitsak, M. *et al.* Identification of influential spreaders in complex networks. *Nature. Phys.* **6**, 888–893 (2010).

[6] Page, L., Brin, S., Motwani, R. & Winograd, T. The pagerank citation ranking: Bringing order to the web. Tech. Rep. 422, Stanford InfoLab (1998).

[7] Morone, F. & Makse, H. A. Influence maximization in complex networks through optimal percolation. *Nature* **524**, 65–68 (2015).

[8] Kempe, D., Kleinberg, J. & Tardos, É. Maximizing the spread of influence through a social network. In *Proc. 9th ACM SIGKDD Int. Conf. on Knowledge Discovery and Data Mining*, 137–146 (ACM, 2003).

[9] Wildt, A. R. & Ahtola, O. *Analysis of covariance*, vol. 12 (Sage Publications, Beverly Hills, CA, 1978).

[10] Burt, R. S. *Structural holes: The social structure of competition* (Harvard university press, Cambridge, MA, 2009).

[11] Blondel, V. D., Guillaume, J.-L., Lambiotte, R. & Lefebvre, E. Fast unfolding of communities in large networks. *J. Stat. Mech. Theor. Exp.* **2008**, P10008 (2008).

[12] Newman, M. E. Analysis of weighted networks. *Phys. Rev. E* **70**, 056131 (2004).

[13] Granovetter, M. The impact of social structure on economic outcomes. *J. Eco. Perspect.* **19**, 33–50 (2005).
